# Cultural contexts of adolescent anxiety: Paradox, ambivalence, and disjuncture

**DOI:** 10.1016/j.ssaho.2024.101081

**Published:** 2024

**Authors:** Sarah Atkinson

**Affiliations:** Institute of Medical Humanities and Department of Geography, Durham University, Lower Mountjoy, DL13 4HL, United Kingdom

**Keywords:** Scale, Context, Individualism, Gender, Environment, Socialisation

## Abstract

The paper offers a critical reading of the dominant ways we frame and understand anxiety in adolescence. These centre on the individual and for the most part limit attention to external and social influences operating at a close scale to the individual. The paper sets out to explore what including perspectives from macro-scale contemporary cultural contexts might reveal and add to how we understand adolescent anxiety. The paper draws on three themes in research on adolescent anxiety: socialisation and development, gender and pressure, environment and uncertainty. Expanding the frame to cultural contexts situates young people's experiences in complex processes from individual to global scales, includes less tangible aspects such as discourse or values, and recognises the importance of experiences of inequalities. The paper proposes that bringing cultural contexts into view reveals a pervasive encounter with paradox, ambivalence and disjuncture in everyday experience through which contemporary adolescent anxiety may be generated and which warrants greater attention. Furthermore, these indicate that some of our most cherished developmental concepts may need a more nuanced understanding of the work they do within the specificities of different cultural contexts.

## 1 Introduction

‘This anxious swirling mess

Of broken thoughts and paralyzed mind.

The impulsive, jerky actions, all corrupting

Inside.’

(Casszilla06, 2022)

‘*The expectations being placed on teens and young adults are high and they can get very overwhelming. … We let that stress and anxiety consume us, or we push it down. … With schoolwork, chores, and jobs we don’t have the time to go outside and take time for ourselves anymore.*’

(Mrussell, 2019)

These words are drawn from publications on *Teen Ink*^1^,^2^, a platform for young writers. Posts about mental health abound and while the majority offer autobiographical reflections on interior affective experience, as in Casszilla06's moving lines, other voices express clear anger at the contexts in which they find themselves. Here, Mrussell details how multiple expectations in all areas of adolescent life can become overwhelming.

The paper takes Mrussell's words as a prompt for looking at adolescent anxiety from a viewpoint beyond the individual, and, as such, starts from a position that treats the rapid increase over recent decades in adolescent anxiety as a social phenomenon. Thinking about anxiety as primarily social foregrounds a suite of considerations related to norms, narratives, inequalities, scales and trends that are here grouped under the umbrella term of cultural context (WHO, 2017). The paper argues that by shifting focus, what comes into view are not only the social, cultural and contextual relations embedding adolescent mental ill-health but the extent that, in combination and across different scales, these generate paradox, ambivalence and disjuncture as characteristics of young people's experiences, identities and future imaginaries. These characteristics of mismatch in everyday experience mostly remain hidden and demand greater research attention.

The paper advances its argument through an essay format, drawing on published empirical studies and conceptual discussion. The exploration limits engagement with cultural variability to the high-income context of the United Kingdom, with further reference to Europe and the USA. The paper briefly summarises the different framings of anxiety in medicine, epidemiology, in psychology, and in philosophy and social science. The paper then explores what might be added to how we frame and understand anxiety by bringing wider social and political critiques of cultural context into dialogue with local scale explorations of anxiety. This draws on and is illustrated through three themes related to anxiety: socialisation; gender; environmental decline. These themes are selected for four reasons: first, they engage recognised social associations with adolescent anxiety; secondly, they foreground different scales - individual, societal, global - at which anxiety is embedded; thirdly, they map onto existing discussions related to anxiety in terms of development, pressure, and uncertainty; lastly, they present unexpected features prompting attention and intimating the omission of something important in current framings. The themes are necessarily selective in this short paper, focussed on probing anomalies and making a preliminary exploration of the implications of bringing different frames and scales into dialogue. The paper thus flags a hidden issue that adds to the agenda for research on anxiety rather than underpinning a new model, approach or set of solutions.

## 2 Framing adolescent anxiety

The reflections by teen writers sit alongside growing alarm of a pre-pandemic global explosion in poor mental health, particularly amongst young people (see WHO, 2018 for the European Region) whose mental health further deteriorated during the pandemic (Racine et al., 2021). Definitions of age categories vary, but fall between 15 and 19 (adolescents) and on up to 25 (adolescents and emerging adults). In this extended age group, anxiety and depression account for over half of all disorders and suicide constitutes the second highest cause of death after road traffic accidents (UNICEF, 2021). Such figures raise considerable concern both for young people themselves and for the known impacts on subsequent life-chances (Mousteri et al., 2019), and the wider stability and productivity of society. At the same time, mental health services are overwhelmed resulting in substantial unmet need. Mental health services largely centre on the individual and predominantly intervene clinically through pharmaceutical treatment (Lagerberg et al., 2019) or psychologically through talking therapies, including counselling, psychoanalysis or cognitive behavioural therapy (CBT). Talking therapies and prevention programmes aim to build individual wellbeing, resilience, and forms of ‘mental health literacy’ that enable adolescents to monitor and manage their own mental health (WHO, 2022a). Despite evident enthusiasm for a range of non-medicalised interventions in everyday settings, and particularly in schools, evidence for efficacy is both limited and weak (Caldwell et al., 2019; Wolpert et al., 2018). Calls for new thinking and new approaches to respond to what is increasingly recognised as a crisis include calls to expand the frames of reference in understanding the processes generating anxiety. Caldwell et al. (2019), for example, reflecting on the limited success of school-based interventions, call for a reorientation in thinking towards multi-systems approaches that address how young lives are situated simultaneously in different scales of influence.

Anxiety is a challenging concept to pin down, variously used as feeling, emotion or condition and mobilised to describe a state that can be everyday, exceptional, beneficial, or pathological. Psychiatry specifies several distinct categories of anxiety disorder, from generalised, social or separation anxiety to more specific expressions of phobias or panic. Adolescents often express these in combination and alongside other mood disorders such as depression or behavioural disruptions such as obsessive-compulsive disorder or eating and body dysmorphic disorders (Cummings et al., 2014; Noyes, 2001). The governing diagnostic guides for mental health disorders are the Diagnostic and Statistical Manual of Mental Disorders (DSM) and the WHO's International Classification of Disease (ICD), in which definition has been based on symptoms rather than a conceptual or etiological understanding since 1980 (DSM-5: APA, 2013; ICD-II: WHO, 2022b). A focus on symptoms side-steps the challenge of defining the term conceptually prompting criticism that the approach risks over-medicalising everyday experience particularly in relation to the range of anxiety, mood, or behavioural disorders (Frances, 2012) and pathologises long-term reasonable worries about concrete challenges (Horwitz, 2012). This medical specification of anxiety locates it as a micro-scale and individual experience that has exceeded the level of what is normal, acceptable, or functionally bearable. Even when reasons for anxiety are identifiable, associated and possible causal factors become secondary to the treatment.

**Fig. 1:**
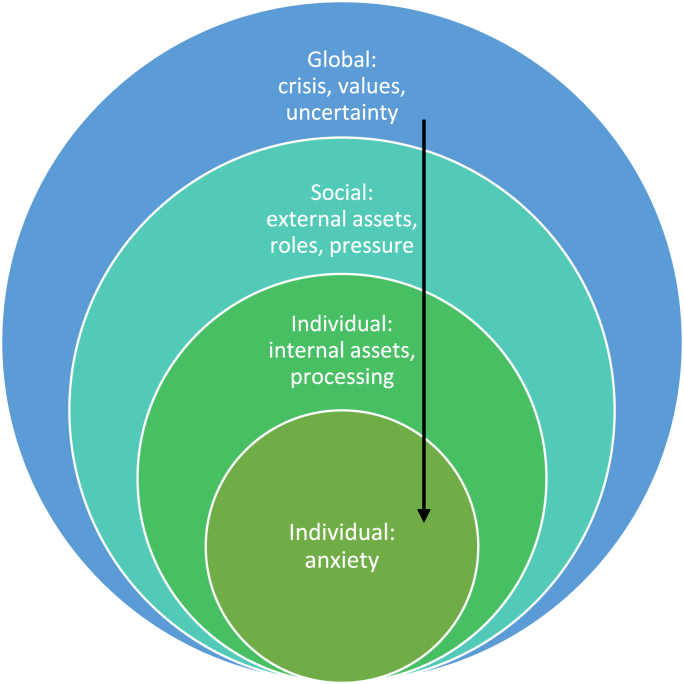
A dominant approach to anxiety: nested scales of uni-directional influence on individual mental health.

Epidemiology, psychology, and the health-facing social sciences show greater recognition of contextual factors in how they frame anxiety. Epidemiological and social research on the determinants of mental ill-health also centre individual experience as the object to be explained but set this explicitly within nested scales of influence from local, proximate influences outwards to global, distant influences. Population increases in adolescent anxiety, however, constitute an aggregation of many individuals responding in a similar mode to common or shared stressors rather than a collective or social phenomenon (Steverson, 2023). Moreover, in practice, while social trends and scales are recognised, more distant and diffuse influences are under-researched or relegated to background descriptors. Psychology offers a range of theoretical resources for exploring and explaining anxiety as individual and social, taking both longitudinal and cross-sectional approaches. Research on key aspects of development and identity formation, for example, draws on internal and external resources or assets in teasing out processes through which adolescents explore, negotiate and commit to identities (see Crocetti et al., 2023; Soares et al., 2024). This focus on process, while rich in explanatory potential, nonetheless remains predominantly engaged with proximate contextual factors. Fig. 1 summarises approaches that centre the individual within nested social and global influences, referencing social determinants and psychological assets approaches. The lack of attention in research on young people's anxiety beyond the meso-scale is stressed by Currie and Morgan (2020) who review the scale of focus of 104 papers on adolescent mental health based on the WHO cross-national study of health behaviour in school-age children. Their striking finding is that half of these papers focus exclusively on the individual, and the vast majority, almost 80%, focus on either individual or micro-scale. There is little attention to relations between factors, reflecting a surprising neglect of multiple association, and of the very few papers that address macro-scale factors, three-quarters do this as cross-national comparisons. It seems, therefore, that even work using large datasets and multiple variables with the potential for exploring contextual differences, neglects analytical attention to wider cultural contexts (Currie & Morgan, 2020).

This is not to deny the insights from such research which identify a range of social factors that may mediate and differentiate adolescent experiences of mental ill-health. Rates tend to increase together with age during adolescence (Kern et al., 2020). Socioeconomic disadvantage is important although economic inequalities show complex patterns of association (Dierckens et al., 2020). Many European countries express a strikingly gendered pattern with rates of anxiety and depression higher amongst young women (Heinz et al., 2020) while young men show higher rates of conduct disorders and higher rates of suicide (UNICEF, 2021).

The relatively rapid deterioration in adolescent mental health demands identification of what it is that has changed over the last two decades in the processes of development, identity formation, and young people's experiences in their everyday environments. Commonly proposed explanatory factors resonate with the young author above, Mrussell. The identification of adolescent-specific determinants for anxiety includes school pressures (Hőgberg et al., 2020), communication stress enabled through smart phones and social media (Beeres et al., 2021; Walsh et al., 2020), technology-mediated sedentary lifestyles, and an increase of body awareness and related dysmorphia (Castelpietra et al., 2022; Heinz et al., 2020). These factors all express themselves in ways that are simultaneously performative, affective, socio-economic, developmental and identity based, and connect and map social trends onto the individual as the site both of experience and expression, and of response and therapy. What is, however, missing in these explanatory models is attention to the macro-scale factors, the relations between them and the processes through which a cultural context connects a diversity of individual and collective experiences. This relative absence of attention to macro-scale factors, and particularly aspects of the political cultural context, is important for at least two reasons. First, a recurrent implication of research on developmental processes is how pre-existing variation in not only disposition but also circumstances shapes and generates further inequalities, including during COVID-19 (see Crocetti et al., 2023; Maiese, 2022; Schweizer et al., 2023). Secondly, the explosion in debilitating anxiety disorders among young people appears to coincide, and potentially reflect, several significant shifts related to ideology, precarity and crisis.

Attention to macro-scale trends of the cultural context is found in what is mostly a separate body of work from philosophy and the more critical political and social sciences. This work positions the manifestation of anxiety in modern society as reflecting a condition of late modernity, a collective malaise, which provokes uncertainties over our imaginings of who we are, how we live and to what purpose (Bauman, 2005; Giddens, 1991). Individual anxiety is framed as an objectless and non-specific feeling or sensation of nameless apprehension, which can connect to an almost limitless set of things, people, events, or ideas (Bergo, 2021; Giddens, 1991). It is, however, also framed as having an affective duality, a feature often overlooked by health-facing research. As such, anxiety can enliven as well as depress, potentially engendering protective and creative actions as well as objectless inertia and paralysis (Bergo, 2021). At societal level, the claims for an unprecedented Age of Anxiety extend from the emergence and convergence of a constellation of uncertainties that range from existential threats to life itself through rapid social, technological, and environmental change to a destabilised sense of the person as impossibly fluid and malleable (Wachs & Schaff, 2020). Reworkings of ideological, cultural, and generational understandings of who we are and how to be characterise the self as constantly becoming through our own will rather than conditioned by our social position (Bauman, 2005; Giddens, 1991; Wachs & Schaff, 2020). This last point is particularly important as responsibility for actualisation and management of this malleable person sits with the individual themself. This effects a circular logic, that is variously virtuous or vicious, in which successful self-management is self-congratulatory while its opposite becomes evidence of personal failure. Recent psychological and experimental research has begun to document the potential toxicity for mental health of the combined expectation of positivity and responsibility for self-management (Dejonckheere et al., 2022). The displacement and replacement of a socially anchored and secure self with one that is malleable and self-creating intersect with other destabilisations of ontological security. Globalisation, and its associated markets, flows and encounters, seems to diminish local attachment and local control over our lives. This sense of powerlessness is further nourished within a pervasive discourse of crisis in response to an ever-growing list of global-scale challenges. This circulating discourse has prompted research, mostly drawing on large data-sets, on specific named crises as potential objects of anxiety and risk. These include political upheavals of post-socialism or Brexit (Cosma et al., 2020; Powdthavee et al., 2019), the 2008 financial crash, environmental deterioration, the recent pandemic, and escalating violence at all scales (Danese et al., 2020; Jia et al., 2020; ONS, 2020; Paleologou et al., 2018; Panchal et al., 2021; Rose et al., 2020; Vakoch and Mickey, 2023). While these crises are named, and, to some extent, known as potential objects of anxiety and risk, they are infused with uncertainty, unpredictability, and uncontrollability. As processes operating at a global scale, they not only threaten existence through their displacement from any clear lines of management or regulation but also reflect a complexity that is difficult to comprehend. These are what Morton (2013) terms ‘hyper-objects’, unable to be fixed as objects of anxiety and threatening to overwhelm and paralyse lives.

Different framings and bodies of evidence thus bring different insights and potential entry-points to understanding adolescent anxiety. There remains, however, a gap, both empirical and conceptual, between approaches primarily focused on individual processes, such as development, identity formation or everyday experiences of overwhelming pressures, and early evidence indicating connections of anxiety to widespread societal change and particularly that associated with uncertainty and existential threat. Two different emerging bodies of work indicate possible bridges between framings focussed on either macro- or more micro-scales. Recent psychological research has started to unpick pathways through which different types of uncertainty may be processed by individuals, for example in relation to reward-sensitivity, risk avoidance and social sensitivity (Schweizer et al., 2023). Alongside this, the growing evidence that adolescent anxiety is associated with perceived environmental threats has prompted social science research through first-person accounts that explicitly connect global processes and individual experiences as bi-directional and multi-faceted (Hickman, 2021). Three thematic examples of adolescent anxiety draw on these different scalar entry-points, specifically development, pressures and uncertainty, to explore the possible intersections of aspects of late modernity and those processes identified at the individual and local social scales.

## 3 Socialised anxiety: Development and identity formation in early years reading

The first theme responds to an unexpected recent emergence of young children's books that explicitly address anxiety. There is a powerful cultural script emerging that positions anxiety as an inevitability of existential experience, including in children, adolescents and young adults. The rise of mental ill-health amongst adolescents has already spawned a distinct fictional genre within young adult literature including engagements with anxiety as an existential experience (Atkinson, 2021a). Literature, as a cultural artefact, can act socially in multiple ways, not only reflecting realities and supporting explorations of own and others experiences but also normalising particular experiences and generating particular expectations of experience. Books, and other forms of communicative media can, therefore, generate creative explorations of anxiety in young people that is ambivalent in acting at different scales in framing anxiety as pathological and a medicalised condition requiring management and recognising the current widespread distress, while simultaneously normalising it as an expectation of the adolescent self and affirming and embedding the view that we live in an age of anxiety.

A radical individualism is now emerging in the characteristic picture and word books available for pre-school and early years readers. Young children's books have, of course, a long history of engaging difficult emotional experiences as a tale unfolds, particularly concrete childhood fears such as the dark, separation, relations, change or several of these in the same story.^3^ Since 2020, however, reflecting impacts on mental health in children from the COVID-19 pandemic, the market for picture and word books has seen a surge in publications that feature medicalised terms, including the word anxiety, in the actual title of the book.^4^ These new offerings tend to share a set of features that mark them as different from earlier engagements with emotions. They are often part of a series of books with a standardised approach to a range of emotions and behaviours^5^ and intentionally marketed as directing good and bad norms of emotional and behavioural management. The blurb, for example, of Mary Nhin's Ninja series (2020–2022) says, ‘ … Ninja Life Hacks is the social emotional learning book series developed to help children learn valuable life skills’ (https://www.ninjalifehacks.tv/). Such ‘skills’ cover characteristic neoliberal normative values in managing conduct and the psychological self (Whitehead et al., 2019) which, in addition to *Anxious Ninja*, include *Hope-, Hard-working-,* and *Growth Mind-Set Ninja.* Supporting young children through emotional uncertainties is an important part of socialisation, but this new genre of word and picture books for younger children constitutes an abrupt shift to using medicalised labelling, treating anxiety as problematic, and, most importantly, socialising the young child into the obligation to affective management through a prescribed set of skills. There is an important difference between previous engagements with early years emotions through storytelling and the recent explosion of what are effectively early years self-help books. In the earlier outings, the story and story-telling remain the primary focus, as evidenced by both the blurb and reviews, which emphasise reader engagement and pleasure. The story, moreover, may carry humour and a certain anarchic narrative even while realising resolution to an emotional challenge. The earlier variants of such books, thus, reassure rather than instruct and, as a result, neither the source nor the resolution of emotions are positioned entirely under the control of the child reader. The macro-scale discourse of self-management sits in a paradoxical tension with our construction of the toddler and young child as needing protection and needing to explore their material and affective worlds. Presenting them with a treatment of their affective world as both problematic and up to them to manage seems riven with possibility for generating further unspecified anxiety through the burden of such early individual responsibility. Moreover, the books reproduce an individualised narrative of anxiety at the expense of locating it even within micro-scale relations and certainly beyond macro- and global-scale relations. As such, anxiety over nameable contextual fears is negated and anxiety over failure, within circumstances of inequality, to meet expectation is likely to be simultaneously amplified. There are publications that succeed in navigating a hybrid position between the old and new and the ambivalences between discourses of self-management and toddler exploration and socialisation. The 2023 winner of the best illustrated book in the Waterstone's Children's Book Prize, Kim Hillyard's ‘*Gretel and the Wonder Mammoth: A Story about Overcoming Anxiety’,* may foreground, and thereby risk normalising, anxiety in the title, but the primary message in the book is not how to exercise emotional self-management but the importance of asking for help.

## 4 Gendered anxiety: Social pressures and expectation

‘*The new standard of test-driven instruction creates immense pressure on students. … School used to stimulate confidence and enthusiasm for many students but now has triggered fear and anxiety.*’

(Nmartone07, 2023)

‘*Of all the strange side effects of the pandemic, I suppose that a boom in the home workout business was inevitable … But the idea that this confusing time should be a time for self-improvement – self-improvement being weight loss – is ridiculous and disgustingly harmful to us …*’

(merryshimwell, 2020)^6^

The privileging of self-management even in early years reflects the dominant cultural context of late modernity. This implicit exhortation to manage and succeed in all areas of life is again central in this second theme of gendered anxiety which is included due to an unexpected finding within the relatively few macro-scale cross country comparisons of adolescent anxiety in Europe. Anxiety shows a strongly gendered expression, but we might not have expected to find this in settings of greater gender equality. The worst rates of deteriorating adolescent mental health, however, tend to be in countries with high income, high gender equality and strong welfare systems, including well developed mental health services, which in Europe includes the Nordic countries and the United Kingdom. This pattern is not what we intuitively expect.

Gendered identities and roles intersect with all the micro-scale factors commonly associated with anxiety in epidemiological studies and are echoed by the two young authors above who both identify as female. School pressures of performance and monitoring impact the mental health of school-age girls more than boys (Högberg et al., 2020). Body dysmorphia associated emotional distress and behavioural expression through eating disorders are more prevalent in young women than men (Heinz et al., 2020). Girls and young women report both higher levels and greater impacts on their mental health of online sexual harassment than their male counterparts (Ståhl & Dennhag, 2020). The correspondence of an increase in anxiety levels with the spread of smart phones has made social media a prime explanatory candidate, a hypothesis further strengthened by the gendered patterning of anxiety as social media tends to be more often used for social evaluation by girls than boys (Beeres et al., 2021).

Gender, as a cultural concept, moves between, infuses, and coheres different scales from features of late modernity through interpersonal interactions to collective ideas of who and how to be. These multiple factors intersect and are embedded in processes beyond the control of the individual, including in cultural norms. Nonetheless, individuals are exhorted and expected to negotiate and manage the everyday experience of these multiple factors, particularly when they express as pressures. The two aspects of school performance and body image focussed on by the teen authors above illustrate how a medical frame may not only miss the importance of cultural context but actually work to exacerbate the situated effects of gendered anxiety. Both authors indicate feelings of pressure to meet expectations set by others and a need to constantly self-monitor action and achievement. The teen authors neatly capture a dominant cultural script that asserts that the pursuit of individual fulfilment and happiness is not just the proper purpose of life, but a promise for those who exercise action and monitoring for self-actualisation. There is a well established critique of the illusory nature of self-actualisation that, as already reflected in the early years picture and word books, effectively predefines what good choices are and treats different choices as moral failure. And, there is little room to excuse failure. A whole industry has developed to support the individual realisation of ‘wellbeing’ through apps to monitor mood, programmes to develop positivity, and endless material goods through which to express self to best effect (see Ehrenreich, 2009).

The promises of self-actualisation, are belied by the everyday experiences in a reality increasingly marked by crisis and precarity, from the micro-scales of employment and housing, through to macro-scales of economy, environment, and the future of life itself. The individualisation of responsibility for wellbeing not only distracts attention from structural inequalities but sets the frame within which those experiencing disadvantage can be blamed for their own condition. This fault-line, connecting different scales in anxiety, plays out through differentiated experiences of gender in the everyday environments of adolescents. Efforts to redress historical biases against young women within education, the workplace and wider society are associated with a barrage of messages and imagery that intentionally champion empowerment, are directed to girls and young women and infuse the spaces of everyday life. Channels for these messages include the school environment, advertising, on-line posts, influencers and commentators, and public celebrities (see Windels et al., 2020 on advertising). The central common thread to empowerment narratives is that girls and young women can exercise agency for self-actualisation to be who they want, do what they want, and have it all. The surge in the levels of anxiety amongst young women may reflect a paradoxical backfiring of this narrative in two ways. First, the exhortation to have it all within the self-management culture of late modernity pressurises young women to demonstrate ability in multiple areas of their life, including schoolwork, appearance, relationships, and other skills. This self-work of constant becoming has been described as constructing a thwarted self who is simultaneously never-quite complete and never-quite adequate (Atkinson, 2021b; Simmonds, 2018). Simmonds, working through young women's own accounts over decades, describes this experience of never-quite good enough as a governing characteristic of the condition of becoming a young woman in late modernity (Simmonds, 2018). This condition of never-quite good enough intertwines with the pressure to perform and the internalisation of self-responsibility, layering demand into an over-burden of expectation. Secondly, this paper takes Simmonds' argument further to propose that the emergence of anxiety is fuelled further by the playing out of these narratives within a context in which not only are young women over-burdened with expectation but in which the promise of an empowered self-actualisation is patently impossible. All societies remain highly differentiated by intersectional relations of class, income, gender, ethnicity and so forth which have very concrete and documented impacts in shaping inequalities of opportunity that inform differentiated life-courses. Moreover, and specific to gender, Western cultural contexts are characterised by conflicting and ambiguous constructions of women in which gains in rights and empowerment are countered by everyday misogyny and violence (Bates, 2014).

At the centre of the observable gendered anxieties, therefore, may be a significant contextual disjuncture, particularly for young women. The internalised script of individual responsibility to define your own worth or shape your own destiny, becomes a hollow soundbite, meaningless beyond performative assertion and masking an invisible mismatch between the promise and the reality. Experiencing this disjuncture and mismatch, this ambivalence of empowerment and disempowerment, on a daily basis by young people in the classroom, the home, online and in potential workplaces, needs framing not as individual experience but as an anxious encounter between cultural contexts of precarity as they intersect with crisis and conflict, and as they are diffracted locally and globally through identity inequalities of sexism, racism, classism and so forth. In this way, a narrative of empowerment that, on the one hand, has potential to furnish a mechanism for containing anxious uncertainties of being, also deepens those uncertainties by disclosing, through a disjuncture with reality, the impossibility of realising the expectations of self-actualisation.

## 5 Eco-anxiety: Uncertainty and existential threat

*‘For the rest of the day I thought about how the future of Earth will be a dark one, covered in plastic and trash. After practice, when I went home, I dreamt of dead fish and turtles choked with plastic, squirrels ran over, dead in the grass.’* (OliverBlount, 2023)^7^

The final theme emerged from a growing body of work exploring adolescent emotions, including anxiety, and activism in relation to environmental degradation. Hogg and colleagues, the developers of a well validated measurement instrument for environment related anxiety, embrace the term ‘eco-anxiety’ to capture the breadth of environmental concerns, including climate change, loss of species diversity, pollution, deforestation and so forth (Hogg et al., 2021). There is growing recognition of young people's eco-anxiety, similar to OliverBlount above, and research has started to explore the complex relations to activism in terms of motivation, individual and collective action and potential mitigation of eco-anxiety through agency (Godden et al., 2021; Kleres & Wettergren, 2017; Knops, 2023; Vamvalis, 2023). This work reveals an ambivalent space in which activism turns confronting the enormous environmental challenges back onto individuals, in this case minors, to take responsibility. Hickman (2020, 2021) highlights how one of the key elements described by adolescents in relation to experiences of eco-anxiety comes from the extent that the adult population and governments appear not to care.

The environment exemplifies Morton's (2013) complex and multiple ‘hyper-object’ that exceeds easy comprehension. It is simultaneously material, organic and atmospheric, and generative, imagined, and discursive. A threat to our environment presents a constant threat to our own existence and, as such, constitutes both anxiety and potential trauma. Environmental crises also disrupt the familiarity of our relation to the planet which, at least in Western cultures, has been characterised by a certitude of human superiority, mastery and entitlement to exploit, often displacing indigenous cultures that respect co-presence with other life-forms (Bednarek, 2021; Buzzell & Chalquist, 2023). The sense of loss, mourning and trauma over environmental damage long expressed by displaced Indigenous peoples (Godden et al., 2021) is now not only articulated by adolescents from Western cultures, but further fuelled by the guilt and ambivalence of being both cause and victim (Hickman et al., 2021). And in contrast to a common tenet of self-care that being in nature benefits wellbeing, Hickman (2020) reports very different adolescent experiences, fuelled by anger, sadness and loss, “*’All I see is lovely trees and that reminds me that we are killing all the trees and then I feel angry and sad, so I won't or can't go there anymore.*’” (p.420).

Mechanisms to address the environmental threat re-enact the wider cultural dominance of a discourse of individual responsibility. Despite the global nature of the threat, the dominant script outside positions of organisational or political power is for individual action in our everyday lives and spaces. While the aggregate effect of large numbers of people reducing their carbon footprint would be significant, the global scale of the structural relations, within which the environmental threat is embedded, operates beyond the reach of micro-scale action. The exercise of individual action, therefore, may serve to stabilise individual eco-anxiety more than address the global challenge. Moreover, while young people may be most concerned about the environment, as minors they are constrained in adopting individual measures and they are the most pessimistic about the potential to take effective action (Stanes et al., 2015). As with gender, positioning responsibility for action onto the individual can generate a feedback loop fuelling rather than mitigating eco-anxiety.

Adolescent environmental concern feeds into a growing discourse of societal tension and disconnect between generations. Again, echoing observations in relation to gender, eco-anxiety and generational disconnect become entangled in parenting and educational styles that display contradictions between a strong ethic of protecting minors and an equally strong ethic to encourage child agency. Adolescents describe their anger with adults, and particularly parents, who variously underplay the risks by telling them not to worry or overplay individual agency by asking them what they might do about it (Hickman, 2020). In the first case, adolescents feel lied to and effectively infantilised as unable to manage the truth. In the second, the opposite is the case and adolescents feel left to solve the problem, despite being minors, for which the adults have abdicated responsibility and ‘over-adulted’ them through encouraging agency. In Europe, such generational tensions over individual responsibilities combine with other societal uncertainties over medium-term futures as regards employment or housing and associated inabilities in meeting established criteria of what it means to be an adult (Holleran, 2020; Thomson & Østergaard, 2020). It also intersects with generational tensions arising through the associated politics of self-management and blame in which some adults perceive adolescent anxiety as reflecting, at best, a lack of resilience, and at worst, generational narcissism, despite the lack of any supporting evidence (Arnett, 2013). As such, eco-anxiety interacts in a wider set of macro-scale uncertainties for and attitudes towards young people that, together, assume a less concrete, nameable, or manageable form. This expression as generational anxiety is made worse by the apparent inability, or even unwillingness, of adults and governments to take action to secure the next generation's future. This suggests to young people that they are growing up in a culture of uncare (Weintrobe, 2021) characterised by a practice of slow violence against them (Nixon, 2011).

The widespread environmental movement by young people, however, explicitly demands action by powerful others as necessary for an ontologically secure future. The benefits that activism effects for anxiety are highly contingent but early research suggests that it is collective, not individual, action that has the potential to relieve adolescent eco-anxiety (Schwartz et al., 2023). The resort by young people to collective activism effectively rejects the positioning of responsibility for environmental security onto individual action through the movement's insistence on radical governmental and cross-national intervention. And in contrast to the dominant treatment of anxiety in the medical context as pathological, the affective force of eco-anxiety is ambivalent in expressing not just a capacity to paralyse daily functioning (Hickman et al., 2021), but also to enliven, motivating activism of young people across different geographical locations (Bright & Eames, 2022).

## 6 Discussion and conclusions: Paradox, ambivalence and disjuncture in adolescent anxiety

The paper set out to explore what viewing adolescent anxiety as a social phenomenon within contemporary cultural contexts might reveal. Different literatures, including from teen authors, and the three themes of anxiety related to socialisation, gender, and environment, foreground entanglements with an often-invisible cultural context. In particular, the unexpected elements in these themes reveal the centrality of various forms of paradox, ambivalence and disjuncture between individualised narratives of choice, agency and empowerment, of expectation and responsibility, and the everyday realities of experience and possibility. Political and psychological commentators have also noted how the very behaviours through which individuals and societies may contain anxiety can simultaneously and paradoxically act to generate anxiety (Dejonckheere et al., 2022; Eberle & Daniel, 2022).

**Fig. 2:**
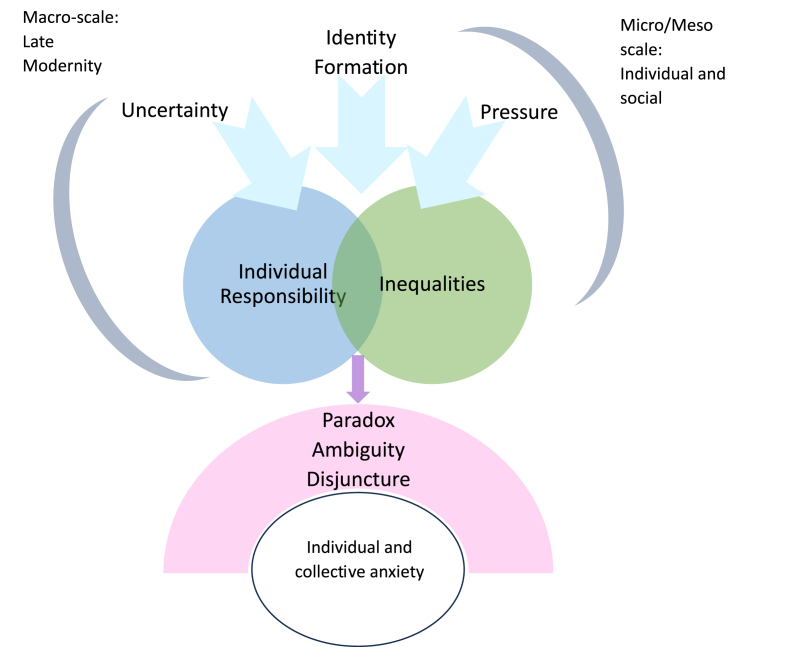
An emergent anxiety: experiences of mismatch across combined scales and influences.

All three themes illustrate how an over-burden of expectation unwittingly lands on young people through a well-intentioned discourse of possibility within a cultural context of neoliberal individualism and responsibility. The promise of social success through self-management in contexts of continuing and entrenched discrimination and inequality not only layers expectations but inevitably generates failure, such that young people are experiencing an everyday disjuncture between what is promised and what is possible. The relationship between adults and young people mediated by cultural context similarly exposes a paradox

between norms of protecting the child and recognising young people's agency. Adult practices that may have good intentions of empowering young people not only feed into the over-burden of expectation but, in relation to the environment, may feel like adult abdication of duty and care. Finally, the ambivalence of anxiety is also only evident from a cultural context perspective. Where the early reader books effectively close down exploration of emotions and what to do with them by prescribing which emotions are good and bad, the theme of eco-anxiety reveals the ambivalence of anxiety in its potential not only to paralyse but also to enliven by enabling its attachment to specific objects and the possibilities to explore what might be done. The importance of the cultural context in infusing, mediating or filtering how other influences are experienced indicates the need to think about scale as less hierarchical and as bi-directional. Fig. 2, drawing on the three themes of the paper, offers an indication of how adolescent anxiety emerges through multiple scales which, in combination and encounter, generate not just overburden, uncertainty or insecure identities but unsettling experiences of paradox, ambivalence and disjuncture.

These examples all expose how a logic that shifts responsibility onto the individual minor or emerging adult may unwittingly convey to young people not only an over-burden of expectation but also a sense of abandonment and lack of care from the adult population. While the contexts and themes explored here are of necessity selective, they serve to prompt particularly difficult questions about the implications of this paper. Activists, including for gender, environmental and child rights, typically champion concepts of empowerment, agency and voice. The themes, however, draw into focus a contextual ambivalence around empowerment and agency. While these may seem incontrovertibly good, their value is always and of necessity contingent on the contexts in which they are mobilised. The paper, then, proposes that it is in the space of such paradox, ambivalence, and disjuncture that adolescent anxiety may be generated and that these warrant much greater research attention.

## CRediT authorship contribution statement

**Sarah Atkinson:** Writing – review & editing, Writing – original draft, Methodology, Investigation, Funding acquisition, Formal analysis, Data curation, Conceptualization.

## Declaration of competing interest

I affirm that, as sole author on the paper, I have no financial or personal interest or belief that could affect my objectivity in constructing the arguments of the paper.

## Footnotes

1 https://www.teenink.com/has run for 25 years, supported by the nonprofit Young Authors Foundation, as the lead in fostering teen writers. It comprises a magazine, book series and website, all devoted entirely to fiction, poetry and non-fiction writing, art, and photos by teens.

2 Casszilla06 Anxiety to fear. December 7, 2022. https://www.teenink.com/poetry/all/article/1174339/Anxiety-To-Fear Mrussell How overwhelming expectations affect teens. March 1, 2019. https://www.teenink.com/opinion/school_college/article/1039772/How-Overwhelming-Expectations-Affect-Teens.

3 For example: *Can't you sleep, little Bear?* Waddell and Firth, 1998; *Baby Owls,* Waddell and Benson, 1994; *Angelina and Alice,* Holabird, 1987; *I Want a Sister*, Ross, 2010; *Dogger*, Shirley Hughes, 1977.

4 For example: Cole, E. *I am stronger than anxiety,* 2021; Hawkins, M.A. *How to tame my anxiety monster,* 2020; Herman, S. *Help your dragon deal with anxiety,* 2018; Hillyard, K. *Gretel the wonder mammoth: a story about overcoming anxiety,* 2022; Laurent, A. *I can help my anxiety,* 2023*;* Nhin, M. *Anxious ninja*, 2020; Treisman, K. *Binnie the baboon anxiety and stress activity book*, 2019.

5 For example: Mary Nhin's Ninja series of over 60 books, 2020–2022; Steve Herman's My Dragon series of 35 books to-date since 2018; Elizabeth Cole's Stronger Than series of eight books to-date since 2020; Adrian Laurent's twelve books on managing ‘Big Emotions’, 2021-23; Sara Koretum's series of five books on medicalised and neurodiverse conditions, 2022, including anxiety, autism, ADHD, anger, and Asperger's.

6 Nmartone07 (2023) Standardized test: an unnecessary source of stress and unrest. April 19, 2023. https://www.teenink.com/opinion/school_college/article/1186881/Standardized-Test-An-Unnecessary-Source-Of-Stress-And-Unrest merryshimwell *The pandemic of home workouts.* June 10, 2020. https://www.teenink.com/opinion/pop_culture_trends/article/1106696/The-Pandemic-Of-Home-Workouts?fbclid=IwAR1YerE6Adxq4Na9PpngiIZ0uVVa9EHgW4UmCfEUb2YzOTdferrz5E3ahDE.

7 OliverBlount A plastic apocalypse. Teen Ink, May 26, 2023. https://www.teenink.com/opinion/environment/article/1191227/A-Plastic-Apocalypse.
